# DSS-induced colitis activates the kynurenine pathway in serum and brain by affecting IDO-1 and gut microbiota

**DOI:** 10.3389/fimmu.2022.1089200

**Published:** 2023-01-26

**Authors:** Li-Ping Zhao, Jian Wu, Wei Quan, Yu Zhou, Hui Hong, Gu-Yu Niu, Ting Li, Shu-Bing Huang, Chen-Meng Qiao, Wei-Jiang Zhao, Chun Cui, Yan-Qin Shen

**Affiliations:** Neurodegeneration and Neuroinjury Laboratory, Wuxi School of Medicine, Jiangnan University, Wuxi, Jiangsu, China

**Keywords:** DSS-induced colitis, tryptophan metabolism, kynurenine pathway (KP), indoleamine-2,3-dioxygenase (IDO), gut microbiota

## Abstract

Accumulative studies suggest that inflammatory bowel disease (IBD) may cause multiple central nervous system (CNS) pathologies. Studies have found that indoleamine-2,3-dioxygenase (IDO, rate-limiting enzyme of the kynurenine (Kyn) pathway) deficient mice were protected from endotoxin induced cognitive impairment, and Kyn administration induced cognitive memory deficits in both control and IDO-deficient mice. However, there is no investigation of the brain Kyn pathway in IBD, thus we investigated whether dextran sulfate sodium (DSS)-induced colitis could cause dysregulation of Kyn pathway in brain, and also in serum. C57BL/6J mice were given drinking water with 2% DSS for 10 consecutive days to induce colitis. In serum, we found significant increase in Kyn and kynurenic acid (Kyna) level, which was regulated by IDO-1 and KAT2 (rate-limiting enzymes of Trp-Kyn-Kyna pathway). Similarly, by analyzing GEO datasets, higher IDO-1 levels in peripheral blood monocytes and colon of UC patients was found. Furthermore, the Kyn pathway was significantly upregulated in the cerebral cortex under the action of IDO-1 after DSS treatment, which ultimately induced the neurotoxic phenotype of astrocytes. To investigate whether gut microbiota is involved in IBD-induced Kyn pathway dysregulation, we performed intestinal flora 16S rRNA sequencing and found that DSS-induced colitis significantly altered the composition and diversity of the gut microbiota. Metabolic function analysis also showed that Tryptophan metabolism, NOD-like receptor signaling pathway and MAPK signaling pathway were significantly up-regulated in the 2% DSS group. A significant association between intestinal flora and Trp metabolism (both in serum and brain) was found by correlation analysis. Overall, this study revealed that DSS-induced colitis causes dysregulation of the Kyn pathway in serum and brain by affecting rate-limiting enzymes and intestinal flora.

## Introduction

Inflammatory bowel disease (IBD) is characterized by chronic inflammation in gastrointestinal tract, including Crohn’s disease (CD) and ulcerative colitis (UC) (1). IBD basically own features of weight loss, diarrhea, abdominal pain and bloody stools (2). The etiology of IBD is still unknow, which is thought to be related to genetic susceptibility, immune dysfunction and microbial imbalance.

Recent years, increasing studies have focused on the influence of IBD on the central nervous system (CNS). Dextran sulfate sodium (DSS) treatment induced depressive-like behavior in mice (3–5), and depression/anxiety is more frequently in IBD patients compared with the general population (6). Furthermore, DSS-induced colitis impaired hippocampal neurogenesis and affected spatial and cognitive memory in mice (7, 8). However, the connection between gut and brain of IBD is still need to be explored.

Kynurenine (Kyn) pathway is the main pathway of tryptophan (Trp) metabolism and is involved in multiple CNS pathologies (9). The main rate-limiting enzyme of Kyn pathway is indoleamine-2,3-dioxygenase (IDO) and tryptophan-2,3-dioxygenase (TDO). IDO deficient mice were protected from cognitive impairment induced by endotoxin, and peripheral injection of Kyn caused recognition memory deficit in both Control and IDO null mice (10). In patients with Alzheimer’s disease, increased IDO-1/TDO expression was co-located with neurofibrillary tangles in hippocampus (11, 12). 1-Methyltryptophan (1-MT, an IDO-1 inhibitor) treatment improved neurotransmitter levels and behavioral parameters in 6-OHDA induced Parkinson’s disease mice while reducing the neuroinflammation marker (TNF-α, IFN-γ and IL-6), oxidative stress and neuronal apoptosis (Caspase-3) (13). These studies suggested that the Kyn pathway is an important mechanism involved in neuropathology, and it is necessary to clarify the change of Kyn pathway under the disease states. However, the effect and mechanism of IBD on brain Kyn pathway has not been explored.

Among pathogenic factors that affect both the gut and brain, the role of intestinal flora cannot be ignored. As an important medium of gut-brain communication, gut microbiota is involved in the pathogenesis of certain CNS diseases such as Parkinson’s disease (14), Alzheimer’s disease (15) and multiple sclerosis (16). Also, intestinal flora is essential for the development of DSS-induced colitis. Germ-free mice showed only minimal inflammation after the induction of colitis by DSS compared with conventional mice (17). Besides, studies have described the alterations in composition and function of the microbiota in IBD patients (18–21). And the alterations were also found in metabolite profiles including bile acids, short-chain fatty acids and Trp metabolites (22). In the intestinal tract, gut microbiota produces a variety of Trp metabolizing enzymes such as Trp decarboxylases, Trp monooxygenase and tryptophanase. These enzymes convert dietary Trp to tryptamine and other molecules, which altered host physiology by reducing available Trp and activated aryl hydrocarbon receptors (23–25). In serum and brain, the effect of gut microbiota on Trp metabolism and the Kyn pathway remain unclear.

Therefore, this study explored the effects of DSS-induced colitis on the Kyn pathway and whether intestinal flora is involved as the mechanism.

## Results

### DSS treatment causes intestinal inflammation and intestinal barrier disruption

**Figure 1:**
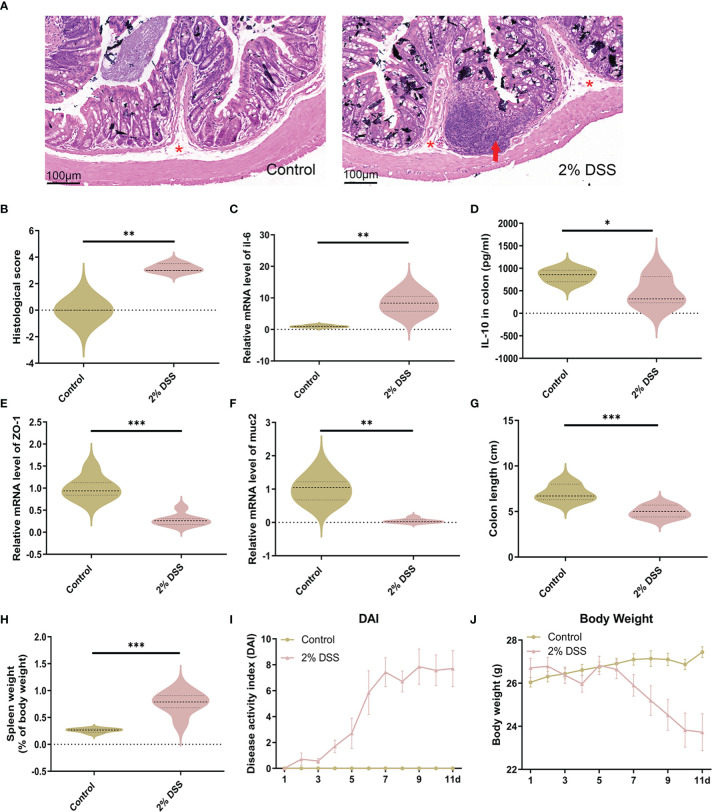
DSS treatment induces intestinal inflammation and intestinal barrier disruption. **(A)** Representative images of colon H&E staining (×25 magnification) showed significant inflammatory cell infiltration after 2% DSS treatment. Red asterisks indicate the location of submucosa, and red arrows indicate extensive infiltration of inflammatory cells in the mucosa. **(B)** Histological injury scores were calculated from inflammatory cell infiltration and tissue damage. **(C)** Il-6 (proinflammatory cytokine) mRNA levels in colon were detected by RT-qPCR. **(D)** Il-10 (anti-inflammatory cytokine) in colon were detected by ELISA. **(E, F)** ZO-1 (intestinal tight junction related molecules) and muc2 (mucins secreted by goblet cells) mRNA levels were detected by RT-qPCR. **(G, H)** Colon length and spleen weight were measured in two groups. **(I)** Evaluation of Disease Activity Index (DAI) during the experiment. **(J)** Change of body weight during the experiment. Data are presented as mean ± SEM, n=3 in **(B)**, and n=5-7 in **C–J**. *P < 0.05, **P < 0.01, ***P < 0.001, P values were determined by the independent samples t test **(B, C, E, G–J)** or Mann-Whitney U test **(D, F)**.

To verify the successful induction of IBD in mice, H&E staining of colon tissue was performed. Significant inflammatory infiltration was found in the mucosal layer after 2% DSS treatment, which lead to a higher histological score in the 2% DSS group compared with the Control group (**Figures 1A, B**). Besides, the expression level of inflammatory cytokines in colon confirmed the inflammation observed in colitis mice. *Il-6* mRNA level (proinflammatory cytokine) was significantly increased 8.3 times (**Figure 1C**), and il-10 (anti-inflammatory cytokines) was significantly decreased by 44.2% in the 2% DSS group compared with the Control group (**Figure 1D**). Colonic inflammation was associated with epithelial integrity and mucus barrier disruption. Significant reduction of *ZO-1* (72.1%) and *muc2* (95.4%) was observed in the 2% DSS group (**Figures 1E, F**), which reflected the damage of mucosal barrier and mucous barrier. Furthermore, colon length and spleen weight were measured as macroscopic evaluations of colonic inflammation. In the 2% DSS group, a 29.4% reduction of colon length was found (**Figure 1G**), and spleen weight was elevated 3.0 times compared with Control group (**Figure 1H**). Disease activity index (DAI) values were significantly increased after DSS treatment due to the increased body weight loss, diarrhea and bleeding (**Figures 1I, J**). Overall, 2% DSS induced significant intestinal inflammation and intestinal barrier damage.

### Colitis causes dysregulation of the Kyn pathway in both DSS administered mice and UC patients

**Figure 2:**
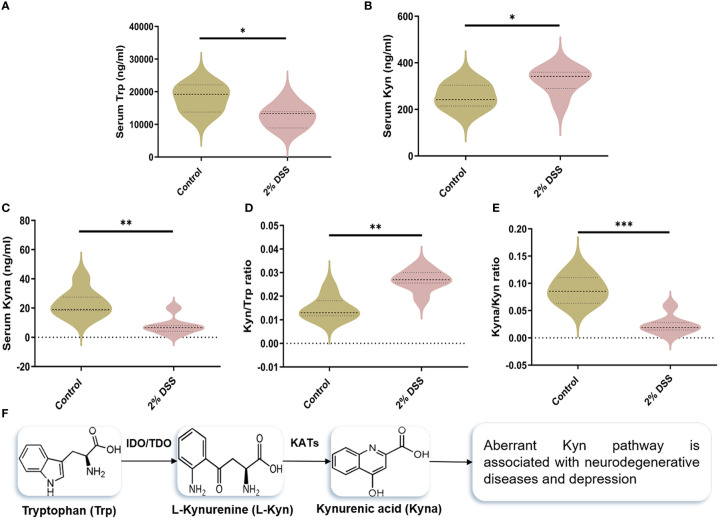
DSS-induced colitis alters tryptophan (Trp) metabolism in serum. **(A–C)** Serum levels of Trp, Kynurenine (Kyn) and kynurenic acid (Kyna) in each group was detected by liquid chromatography-mass spectrometry (LC-MS) assay. **(D)** Kyn/Trp ratio indicated elevated efficiency of Trp metabolism into Kyn. **(E)** Kyna/Kyn ratio indicated decreased efficiency of Kyn metabolism into Kyna. **(F)** Representation of Trp catabolism along the Kyn pathways. Data are presented as mean ± SEM, n=7. *P < 0.05, **P < 0.01, ***P < 0.001, P values were determined by the independent samples t test **(A, B, E)** or Mann-Whitney U test **(C, D)**.

To investigate whether 2% DSS induced colitis affected the metabolism of serum Trp, we detected the levels of Trp and its metabolites (Kyn and Kyna) in serum. The level of serum Trp was decreased by 30.6% in 2% DSS group compared with Control group (**Figure 2A**). Kyn level in serum was increased 1.3 times (**Figure 2B**), while Kyna was decreased by 66.9% after DSS treatment (**Figure 2C**). Moreover, Kyn/Trp ratio and Kyna/Kyn ratio confirmed this effect. Kyn/Trp ratio was significantly elevated 1.8 times in 2% DSS group (**Figure 2D**), which reflected increased efficiency of Trp metabolism into Kyn. Kyna/Kyn ratio was decreased by 73.2% in 2% DSS treatment group (**Figure 2E**), indicated the reduction of Kyn metabolism into Kyna. These results indicated that DSS treatment significantly changed the levels of Trp and its metabolites in serum.

**Figure 3:**
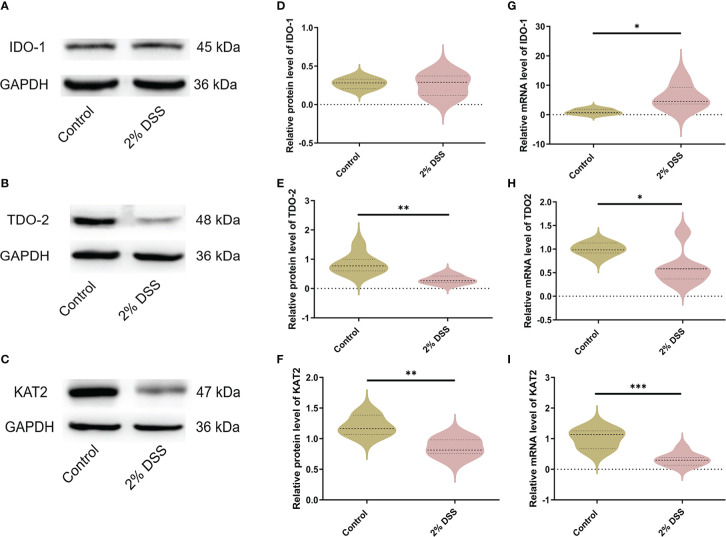
DSS-induced colitis alters metabolic enzyme levels of Kyn pathway in liver. **(A–C)** Representative western blot of IDO-1, TDO-2 and KAT2 expression in liver. **(D–F)** Intensity of bands was quantified with Image J software and quantitative data for IDO-1, TDO-2 and KAT2 following normalization to GAPDH. **(G–I)** IDO-1, TDO-2 and KAT2 mRNA levels in liver were detected by RT-qPCR. Data are presented as mean ± SEM, n=6-7. *P < 0.05, **P < 0.01, ***P < 0.001, P values were determined by the independent samples t test **(D–F, G, I)** or Mann-Whitney U test **(H)**.

The levels of rate-limiting enzymes are the direct factor affecting Trp metabolites. IDO-1 and TDO-2 are the main rate-limiting enzymes that metabolize Trp into Kyn, and KAT2 is rate-limiting enzyme that metabolize Kyn into Kyna (**Figure 2F**). IDO-1, TDO-2 and KAT2 are all expressed in liver cells. Therefore, we detected the levels of IDO-1, TDO-2 and KAT2 in the liver to explore the causes of Trp metabolite changes. The protein level of IDO-1 in liver did not achieve significance between the two groups (**Figures 3A, D**), while a significant increase (5.9 times) of *IDO-1* mRNA level was observed in 2% DSS group compared with Control group (**Figure 3G**). TDO-2 decreased by 64.1% and 41.2% respectively in protein level (**Figures 3B, E**) and mRNA level (**Figure 3H**) in 2% DSS group compared with Control group. This indicated that the increase in Kyn level was not due to elevated expression of IDO-1 and TDO2. Besides, KAT2 decreased by 28.8% and 67.0% respectively in protein level (**Figures 3C, F**) and mRNA level (**Figure 3I**) in 2% DSS group compared with Control group, which suggested that decreased Kyna level might be contributed to reduced KAT2 protein. In brief, the alteration of Trp-Kyn-Kyna pathway in serum was partly regulated by rate-limiting enzymes.

**Figure 4:**
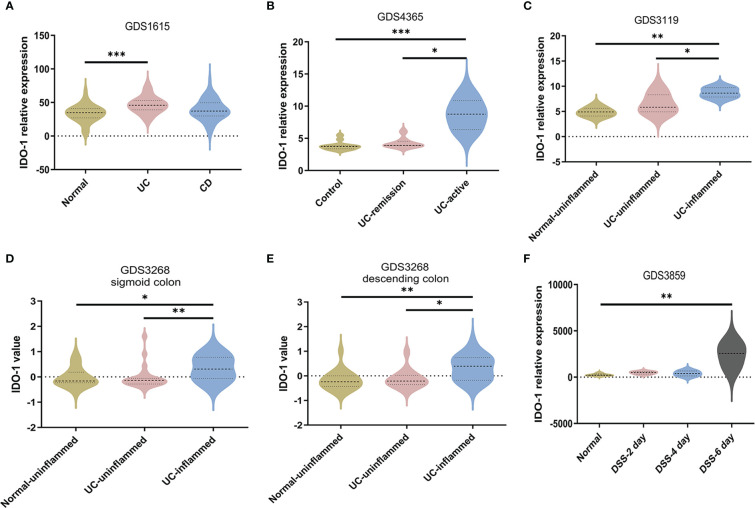
Upregulation of IDO-1 in peripheral blood mononuclear cells and colitis tissues. **(A)** IDO-1 expression in peripheral blood mononuclear cells from UC patients and CD patients were collected from GEO database (GDS1615, n=42 for normal group, n=26 for UC group and n=59 for CD group). **(B)** IDO-1 expression in control colon tissues and colonic tissues from UC patients (remission or active stage) were collected and analyzed from GDS4365 (n=13 for Control group, n=8 for UC-remission group and n=15 for UC-active group) **(C–E)** IDO-1 expression in uninflamed normal colonic tissues and colonic tissues from UC patients (uninflamed or inflamed colon) were collected and analyzed from GDS3119 (n=5 for Normal-uninflamed group, n=13 for UC-uninflamed group and n=8 for UC-inflamed group) and GDS3268 (sigmoid colon: n=24 for Normal-uninflamed group, n=25 for UC-uninflamed group and n=32 for UC-inflamed group; descending colon: n=22 for Normal-uninflamed group, n=15 for UC-uninflamed group and n=19 for UC-inflamed group). **(F)** IDO-1 expression in normal colonic tissues of C56BL/6J mice and 3% DSS-treated mice were collected and analyzed from GDS3859 (n=5 for Normal group, n=6 in DSS-2 day, DSS-4 day and DSS-6 day groups). Data are presented as mean ± SEM. *P < 0.05, **P < 0.01, ***P < 0.001, P values were determined by one-way ANOVA with *post hoc* comparisons of Bonferroni **(A, C)** or Kruskal-Wallis test **(B, D–F)**.

To further confirm the dysregulation of Kyn pathway in IBD patients, we analyzed four NCBI Gene Expression Omnibus (GEO) datasets (GDS1615, GDS4365, GDS3119, GDS3268) containing expression profiles of peripheral blood mononuclear cells and colonic mucosa from IBD patients, and one GEO dataset (GDS3859) containing expression profile of colon tissue from DSS-induced mouse colitis. We found that *IDO-1* mRNA levels were significantly higher in peripheral blood mononuclear of UC patients but not CD patients compared with the normal group, which indicated that colitis induced the activation of IDO-1 in immune cells (**Figure 4A**). In colonic mucosa of UC patients, *IDO-1* expression was significantly higher in UC-active/UC-inflamed patients compared with Control or UC-remission/UC-uninflamed patients, which reflected that colitis induced the activation of IDO-1 in colon (**Figures 4B–E**). Besides, colonic *IDO-1* expression was significantly increased after 6 days treatment of DSS compared with normal group in C57BL/6J mice aged 12~14 weeks old (**Figure 4F**). Collectively, these results demonstrated the increase of *IDO-1* in UC patients and colitis mice, indicated upregulation of Kyn pathway.

### DSS-induced colitis activates Trp-Kyn pathway in the cerebral cortex by regulating IDO-1 expression

**Figure 5:**
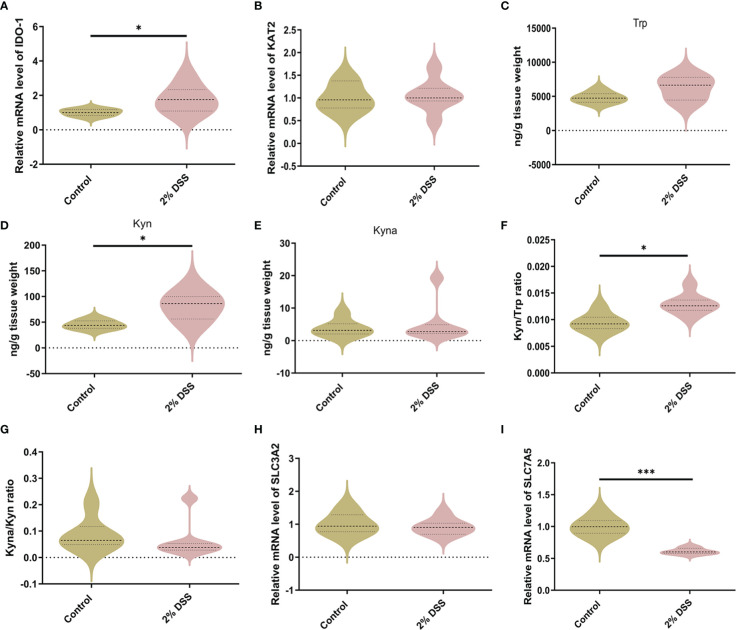
DSS-induced colitis alters Kyn pathway of Trp metabolism in the cortex. **(A, B)** Trp metabolic enzymes levels of IDO-1 and KAT2 in cortex were detected by RT-qPCR. **(C–E)** Trp metabolite levels including Trp, Kyn and Kyna in cortex were detected by LC-MS. **(F)** Kyn/Trp ratio indicated elevated efficiency of Trp metabolism into Kyn in cortex. **(G)** Kyna/Kyn ratio indicated unchanged efficiency of Kyn metabolism into Kyna in cortex. **(H, I)** SLC3A2 and SLC7A5 (L-type amino acid transporter LAT1 subunits) mRNA levels in cortex were detected by RT-qPCR. Data are presented as mean ± SEM, n=6-7. *P < 0.05, ***P < 0.001, P values were determined by the independent samples t test **(A–D, F, H, I)** or Mann-Whitney U test **(E, G)**.

Subsequently, we explored the effects of DSS-induced colitis on Trp metabolism in the cerebral cortex. Compared with Control group, *IDO-1* mRNA level was significantly increased 1.8 times after 2% DSS treatment (**Figure 5A**), while the level of *KAT2* was unchanged in 2% DSS group (**Figure 5B**). As a result, Kyn level was elevated in 2% DSS group compared with Control group, while Trp and Kyna did not achieve significance in the two groups (**Figures 5C–E**). This suggested that the level of Kyn and Kyna were under the regulation of IDO-1 and KAT2. Kyn/Trp ratio also showed that the efficiency of Trp metabolism to Kyn was improved (**Figure 5F**), which was consistent with elevated levels of IDO-1 and Kyn. The Kyna/Kyn ratio did not achieve significance between Control and 2% DSS group (**Figure 5G**), which reflected Kyn-Kyna pathway was not affected by DSS treatment. The transfer of Kyn from blood to brain is mediated by L-type amino acid transporter 1 (LAT1). We detected *SLC3A2* (4F2hc heavy subunit) and *SLC7A5* (CD98 light subunit) of LAT1 transporter subunits at the mRNA levels. There was no change in *SLC3A2* between two groups (**Figure 5H**), while *SLC7A5* was significantly decreased by 38.8% in 2% DSS group compared with Control group (**Figure 5I**). Therefore, we inferred that elevated Kyn in the cerebral cortex was mainly derived from IDO-1-mediated local synthesis rather than translocation from the blood.

### DSS-induced colitis causes neurotoxic phenotype of astrocytes but not microglia at mRNA levels

**Figure 6:**
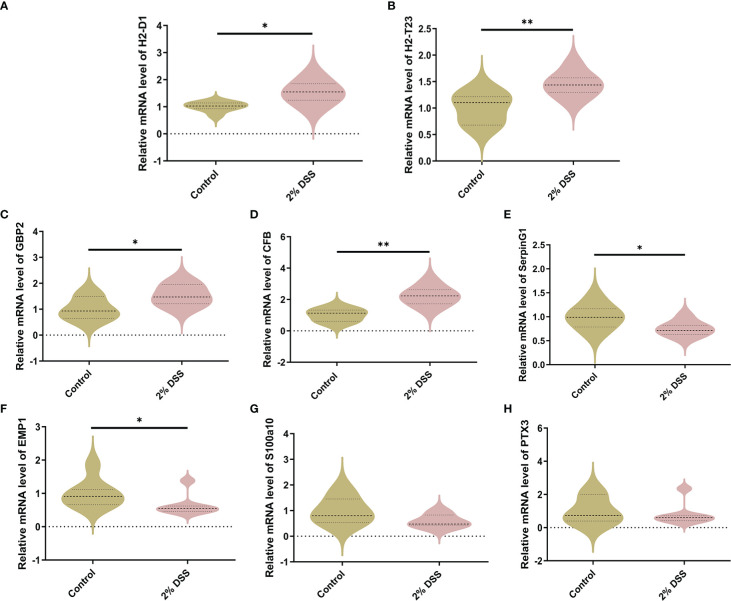
DSS-induced colitis induces neurotoxic phenotype (A1) of astrocyte. **(A–E)** A1-specific markers of astrocyte (H2-D1, H2-T23, GBP2, CFB and SerpingG1) were detected by RT-qPCR in cortex. **(F–H)** A2-specific markers of astrocyte (EMP1, S100a10 and PTX3) were detected by RT-qPCR in cortex. Data are presented as mean ± SEM, n=5-7. *P < 0.05, **P < 0.01, P values were determined by the independent samples t test **(A–F)** or Mann-Whitney U test **(G, H)**.

Previous studies have shown that DSS-induced colitis caused cortical inflammation, as demonstrated by significant elevation of proinflammation cytokines (il-6 and tnf-α) and increased microglial activation in cortical tissue (26). This suggested that the cerebral cortex is a brain target affected by colitis. Furthermore, Kyn administration also induced brain infiltration of proinflammatory monocytes and astrocytic activation (27, 28). Therefore, to further investigate whether Kyn elevation caused by colitis can alter astrocyte and microglia subtypes, we detected the expression levels of astrocyte and microglia markers. In A1-specific markers of astrocyte, *H2-D1*, *H2-T23*, *GBP2* and *CFB* mRNA levels were significantly increased by 51.9%, 44.8%, 52.9% and 121.8% (**Figures 6A–D**), while *SerpinG1* was decreased by 24.8% in 2% DSS group compared with Control group (**Figure 6E**), which indicated the expression of A1-specific markers of astrocyte was elevated in colitis group. In A2-specific markers of astrocyte, *EMP1* was decreased by 35.7% in 2% DSS group compared with the Control group (**Figure 6F**), while there was no significant difference in *S100a10* and *PTX3* between the two groups (**Figures 6G, H**). These data suggested that astrocytes in colitis group were driven to an A1 phenotype.

**Figure 7:**
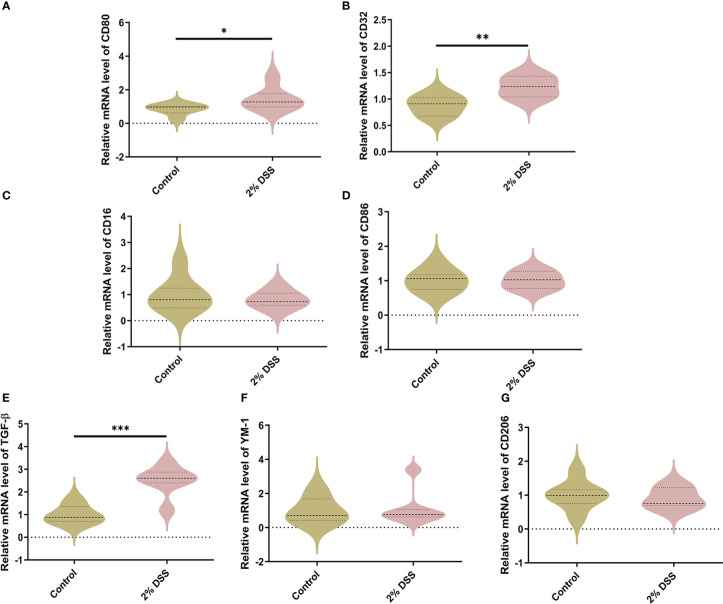
DSS-induced colitis alters microglial phenotypes related gene expression in cortex. **(A–D)** M1-specific markers of microglia (CD80, CD32, CD16, and CD86) were detected by RT-qPCR in cortex. **(E–G)** M2-specific markers of microglia (TGF-β, YM-1 and CD206) were detected by RT-qPCR in cortex. Data are presented as mean ± SEM, n=6-7. *P < 0.05, **P < 0.01, ***P < 0.001, P values were determined by the independent samples t test **(A–D, G)** or Mann-Whitney U test **(E, F)**.

For M1-specific markers of microglia, the mRNA levels of *CD80* and *CD32* were significantly elevated by 44.3% and 22.9% respectively in 2% DSS group (**Figures 7A, B**), while the levels of *CD16* and *CD86* did not achieve significance between 2% DSS group and Control group (**Figures 7C, D**). For M2-specific markers of microglia, the mRNA levels of *TGF-β* were significantly increased by 151.0% in 2% DSS group compared with Control group (**Figure 7E**), while there is no significant difference in *YM-1* and *CD206* between the two groups (**Figures 7F, G**). There was no significant change in M1/M2 phenotypes transition of microglia cells. Overall, these results suggested that DSS-induced colitis caused neurotoxic subtype of astrocytes, which may be attributed to increased levels of Kyn in the cerebral cortex.

### DSS-induced colitis alters beta diversity and composition of intestinal flora

**Figure 8:**
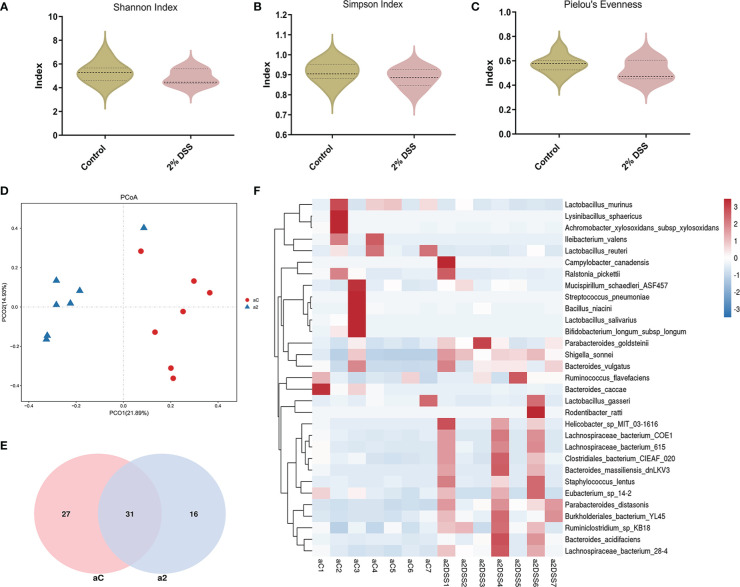
DSS-induced colitis significantly changed the beta diversity and species abundance of gut flora. **(A–C)** Analysis of alpha diversity showed no significant difference in Shannon index, Simpson index and Pielou’s evenness between Control group and 2% DSS group. **(D)** Principal coordinate analysis (PcoA, reflecting beta diversity) indicated lower sample composition similarity between two groups. **(E)** Venn diagram showed common and specific species of gut flora in Control group and 2% DSS group. **(F)** Heatmap showed the relative abundance of gut microbiota at the species level in Control group and 2% DSS group. Data are presented as mean ± SEM, n=7. P values were determined by the independent samples t test **(A–C)**. aC, a2 at **(D–F)** represented Control group and 2% DSS group.

**Figure 9:**
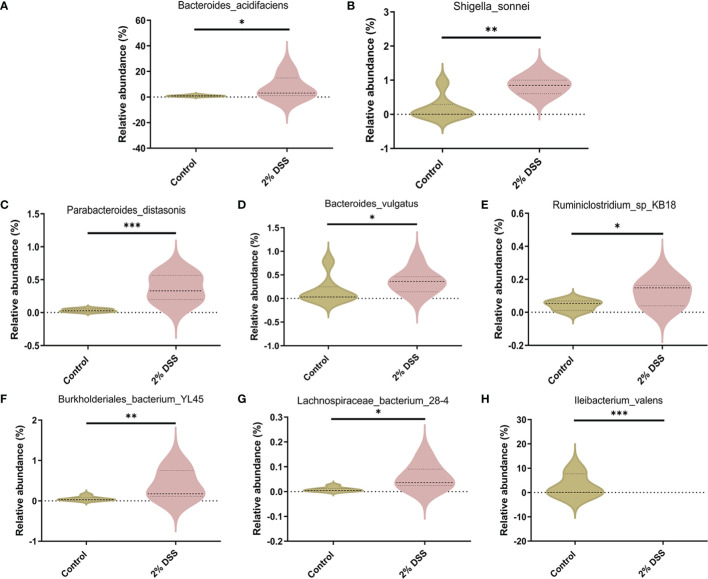
Species with significant abundance difference after 2% DSS treatment compared with the Control group. **(A–H)** At the species level, 2% DSS treatment significantly increased the abundance of *Bacteroides_acidifaciens*, *Shigella_sonnei*, *Parabacteroides_distasonis*, *Bacteroides_vulgatus*, *Ruminiclostridium_sp_KB18*, *Burkholderiales_bacterium_YL45* and *Lachnospiraceae_bacterium_28-4* **(A–G)**, and decreased the abundance of *Ileibacterium_valens* **(H)**. Data are presented as mean ± SEM, n=7. *P < 0.05, **P < 0.01, ***P < 0.001, P values were determined by the independent samples t test **(C, E, G)** or Mann-Whitney U test **(A, B, D, F, H)**.

Several studies have shown that gut microbiota (such as *Akkermansia muciniphila* and *Parabacteroides distasonis*) is involved in the regulation of Trp metabolism (29–31). To investigate the characters of the gut flora after 2% DSS treatment, 16S rRNA sequencing was performed with the colon contents. Alpha diversity indices including Shannnon Index, Simpson Index and Pielou’s Evenness did not achieve significance between 2% DSS group and Control group (**Figures 8A–C**), which reflected unaffected species diversity and evenness. Principal coordinate analysis (PcoA) indicated a lower similarity of sample composition between the two groups (**Figure 8D**). Furthermore, Venn diagram showed 27 and 16 unique species respectively in Control group and 2% DSS group. And 31 common species were found in both groups (**Figure 8E**). The relative abundance of 31 common species was shown in the heatmap (**Figure 8F**), and only 8 common species achieved significance between Control group and 2% DSS group. The abundance of *Bacteroides_acidifaciens*, *Shigella_sonnei*, *Parabacteroides_distasonis*, *Bacteroides_vulgatus*, *Ruminiclostridium_sp_KB18*, *Burkholderiales_bacterium_YL45* and *Lachnospiraceae_bacterium_28-4* were significantly increased, and the abundance of *Ileibacterium_valens* was significantly decreased in 2% DSS group compared with Control group (**Figure 9**). These data reflected that DSS-induced colitis altered the diversity and composition of gut flora.

### Intestinal microflora is involved in Trp metabolism

**Figure 10:**
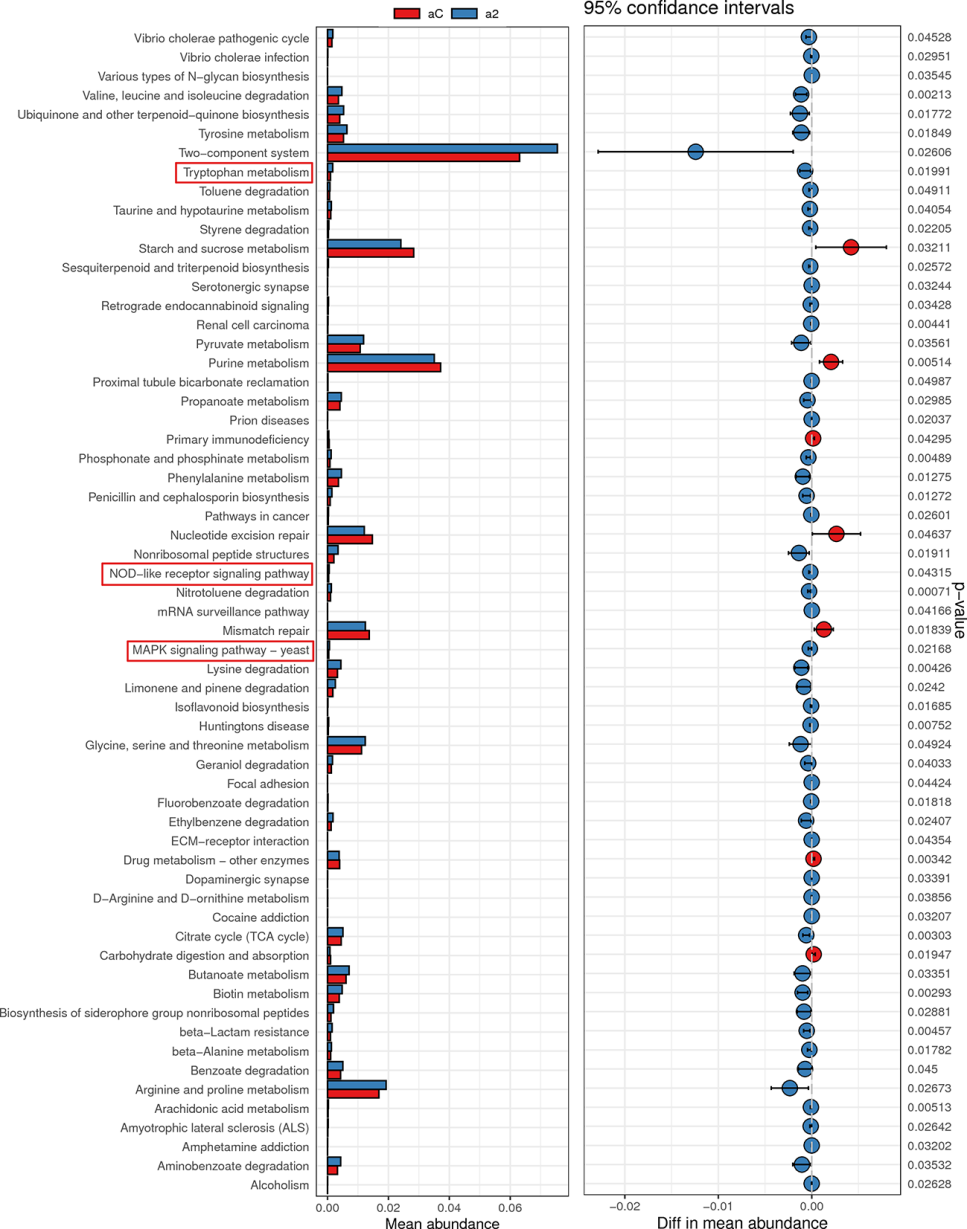
Metabolic function prediction of intestinal flora in Control group and 2% DSS group. Tax4Fun analysis was used to predict KEGG metabolic pathways. Relative abundance of Tryptophan metabolism, NOD-like receptor signaling pathway and MAPK signaling pathway were significantly increased in 2% DSS group compared with Control group.

**Figure 11:**
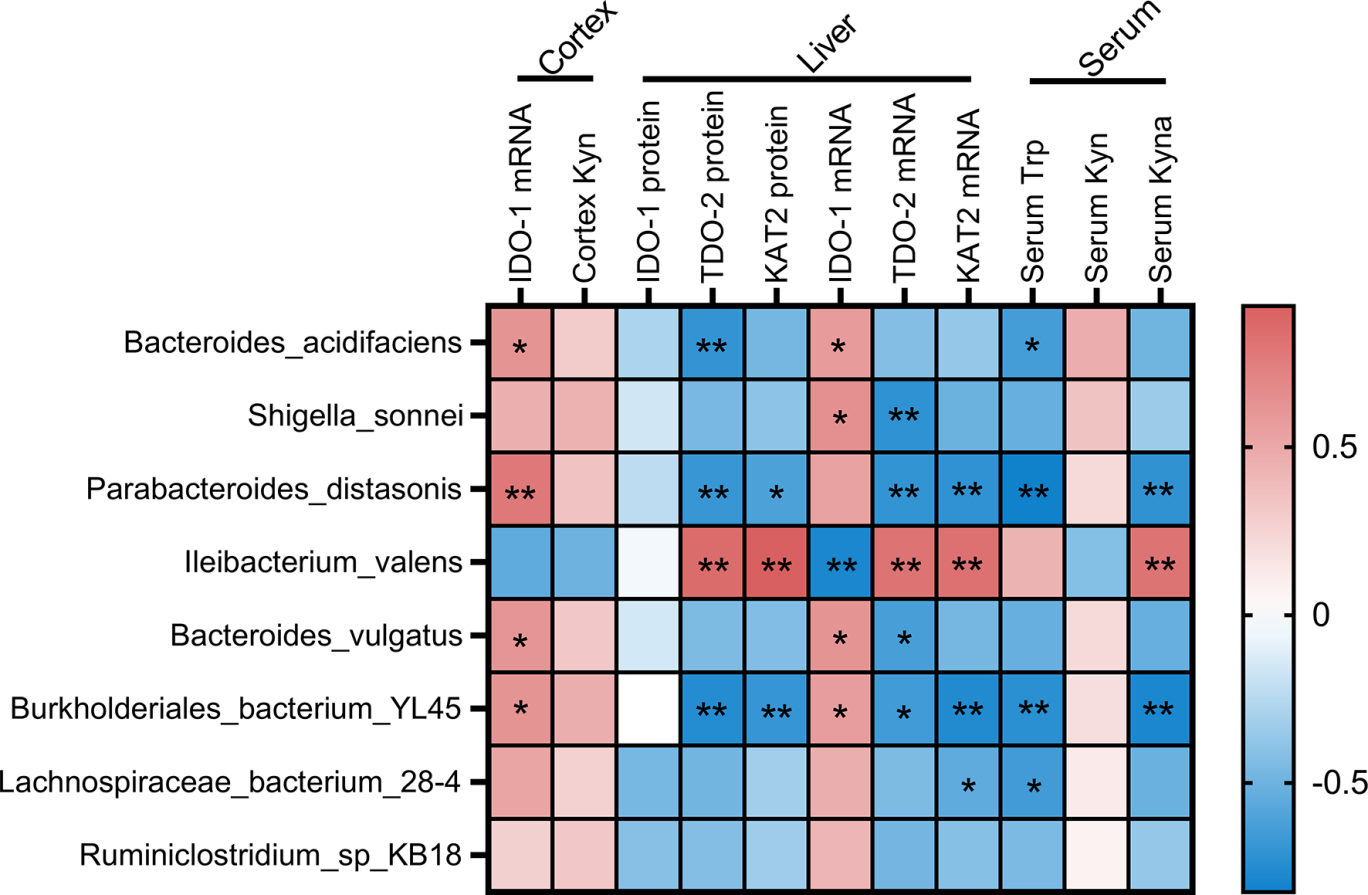
Associations of the intestinal flora and Trp metabolism. The intestinal flora listed in the figure were species with significant differences in abundance between Control and 2% DSS group. Pearson’s correlation coefficient was calculated for normally distributed data, and the Spearman’s correlation coefficient was calculated for non-normal data. Significant associations were displayed with asterisk (*P < 0.05, **P < 0.01).

Given the change of Trp metabolism is related to intestinal flora, metabolic functions of the gut microbiota were assessed by PICRUSt2 based on 16S rRNA metagenomics data. Several functional pathways in the microbiome of 2% DSS group were found elevated, including Tryptophan metabolism, NOD-like receptor signaling pathway and MAPK signaling pathway (**Figure 10**). In addition, species with significant differences in abundance between the Control group and 2% DSS group were significantly associated with rate-limiting enzymes and metabolites of the Trp-Kyn-Kyna pathway (**Figure 11**), which indicates the regulatory effects of gut flora on Trp metabolism in the serum and cerebral cortex. Specifically, we found that several species were significantly correlated with the levels of IDO-1/TDO-2 in cortex and liver, but not with Kyn, suggested that gut microbiota may affect Kyn by regulating the level of IDO-1. The species involved in this process included: *Bacteroides_acidifaciens*, *Shigella_sonnei*, *Parabacteroides_distasonis*, *Ileibacterium_valens*, *Bacteroides_vulgatus* and *Burkholderiales_bacterium_YL45*. In addition, several species including *Parabacteroides_distasonis*, *Ileibacterium_valens* and *Burkholderiales_bacterium_YL45* were significantly correlated with both KAT2 in liver and Kyna in serum, indicating that intestinal flora may regulate Kyna level in serum directly or by affecting KAT2.

## Discussion

This study investigated the effect of DSS-induced colitis on the Kyn pathway of Trp metabolism in the brain and serum, and the role of gut microbiota. In DSS-induced colitis mice, we found a significant increase in serum Kyn level and a significant decrease in serum Kyna level, which was mediated by the rate-limiting enzymes IDO-1 and KAT2. To verify this finding, we analyzed GEO datasets and found that IDO-1 levels were significantly upregulated in peripheral blood monocytes and colon of UC patients, indicated the dysregulation of Trp-Kyn pathway. Similarly, the Kyn pathway was significantly upregulated in the cerebral cortex under the action of IDO-1 after DSS treatment, which ultimately induced the neurotoxic phenotype of astrocytes. Furthermore, DSS-induced colitis altered gut microbiota composition and diversity. Metabolic functions analysis based on 16S rRNA metagenomics data found that Trp metabolism pathway was significantly elevated in 2% DSS group compared with the Control group. And significant correlation was found between gut microbiota and the Trp-Kyn pathway, implied the regulatory role of gut microbiota on Trp metabolism in serum and cerebral cortex.

In this study, we found that DSS-induced colitis caused a significant decrease in serum Trp content and increase in Kyn content, which was consistent with previous studies (32–34). The decrease in serum Trp content may be due to reduced food intake (the main source of Trp) in colitis mice (35). The elevation of Kyn content was mainly attributed to the decrease of its consumption. We detected the downstream product (Kyna) of Kyn and the rate-limiting enzyme (KAT2), and found that both of them were significantly reduced. In addition, the protein level of IDO-1, a rate-limiting enzyme for Kyn synthesis, did not achieve significance between Control and 2% DSS group, while the *IDO-1* mRNA level was significantly increased after DSS treatment. The previous study found that only 40% of the protein variation could be explained by known mRNA abundance, and most part of the variation in proteins could be attributed to post-transcriptional regulation and measurement noise (36). We speculated that the inconsistent changes of *IDO-1* mRNA and protein levels were probably due to post-transcriptional regulation.

The effect of colitis on the Kyn pathway in the brain has not been reported. In this study, we found that the Kyn pathway was significantly activated in the cerebral cortex after DSS-induced colitis, which was manifested by the significant elevation of Kyn and *IDO-1*. Approximately 60% of the Kyn in the brain comes from the periphery because it can cross the blood-brain barrier *via* the large neutral amino acid transporters (LATs) (37, 38). We detected two subunits of LAT1 and found that only *SLC7A5* was significantly decreased in 2% DSS group, which implied that the increased Kyn in cerebral cortex is mainly due to *IDO-1* mediated local elevation rather than from peripheral blood. Kyn in the brain can be metabolized by astrocytes and microglia to downstream products such as 3-hydroxykynurenine and Kyna, and can also affect cell status (39). Kyn (48 μM) treatment reduced LPS-induced expression of proinflammatory cytokines (*IL-1β*, *IL-6* and *TNF-α*) in BV2 microglia (39). Besides, Kyn treatment resulted in up-regulation of NLRP2 protein expression in primary astrocytes in a concentration-dependent and time-dependent manner, which indicated the proinflammatory effect of Kyn (28). In our study, DSS-induced colitis causes neurotoxic phenotype of astrocytes but not microglia in the cerebral cortex, which may be attributed to elevated Kyn. However, the effect of DSS-induced elevation of Kyn on brain function remains unclear and needs to be further explored.

Some papers suggested gut microbiota is involved in Trp metabolism. For instance, fecal microbiota transplants from IDO-1-deficient mice directly attenuated the severity of DSS-induced colitis in IDO-1^+/+^ mice (40), which suggested that intestinal flora regulated the severity of colitis in Trp metabolism-dependent manner. In addition, fecal microbiota transplants from schizophrenic patients activated Kyn-Kyna pathway of Trp metabolism (41), which demonstrated the involvement of gut microbiota in Trp metabolism. In this study, we also found that DSS-induced colitis caused significant changes in the composition of gut microbiota compared with Control group, which is supportive to above-mentioned studies (42–44). In addition, we investigated the relationship between gut microbiota and Trp metabolism. PICRUSt analysis found that Trp metabolism-related microbial gene functions were higher in 2% DSS group. This is consistent with our findings in serum and brain, suggested that gut microbiota played an important role in Trp metabolism. Moreover, correlation analysis showed a significant association between intestinal flora and the Trp-Kyn-Kyna pathway of Trp metabolism. The previous study has shown that JAK-STAT signaling pathway and melatonin-JNK-FoxO1 pathway may be the regulators of IDO-1 expression (45). Our study found NOD-like receptor signaling pathway and MAPK signaling pathway of microbial gene functions were activated after DSS treatment, which implied that these two pathways may be the mechanism of gut microbiota affecting Trp metabolism in future studies.

## Materials and methods

### Animals

Male C57BL/6 mice (aged 10 weeks, Vital River Laboratory Animal Technology, Pinghu, China) were used in this study. Animals were kept in specific pathogen-free environment (12h light/12h dark cycle) with regulated temperatures (24 ± 2.0°C) and humidity (55 ± 10%). Water and food were available ad libitum (Jiangsu Xietong Organism, China). Mice were acclimated for one week before the experiments. All animal experimental procedures were approved by the Animal Ethics Committee of Jiangnan University.

### Establishment of DSS-induced colitis model

The mice were randomly allotted to two groups: Control group and 2% DSS group (n = 7 in each group). The Control group received sterile tap water as drinking water. Experimental colitis was induced by adding 2% DSS (36,000-50,000 Da, MP Biomedicals, Solon, USA) in drinking water for 10 days (from day 1 to day 10). Body weights, diarrhea (stool consistency) and rectal bleeding were daily recorded. All mice were sacrificed after being deeply anesthetized by isoflurane on day 11. Tissues (colon, liver and cerebral cortex), blood and colonic contents were collected. After centrifugation (4°C, 3000 rpm, 15 minutes), serum was separated from the blood.

### Haematoxylin and Eosin staining and histopathological analysis

Colonic tissue (proximal colon to the rectum) was collected and fixed in 4% paraformaldehyde at 4°C. Following embedded in paraffin, the colon was sectioned transversely (5 μm in thickness) and stained with H&E (C0105S, Beyotime, Shanghai, China). The histological changes were recorded under LuxScan 10K/B (CapitalBio, Beijing, China). As described previously, two parameters were used to calculate the histological damage score: tissue damage (score: 0-3) and infiltration of inflammatory cells (score: 0-3) in a double-blind fashion (46). Three sections obtained from each of three sites at 100 μm distance were evaluated, and mice were scored individually with each score representing the mean of nine sections (47).

### Quantitative real-time PCR analysis

According to the manufacturer’s instructions, total RNA from colon and cerebral cortex was extracted using Total RNA Extraction Reagent (R401-01, vazyme, Nanjing, China). Then, the total RNA was reverse transcribed into cDNA using HiScript III RT SuperMix for qPCR (R323-01, vazyme, Nanjing, China). PCR was performed using ChamQ Universal SYBR qPCR Master Mix (Q711-00, vazyme, Nanjing, China). Primers used are listed in **Table 1**. The 2^-ΔΔCt^ method was used for mRNA quantification. The mRNA expression of target gene was normalized to GAPDH expression.

**Table 1:** Sequences of the gene-specific primers.

Gene ID	Gene	Forward primer (5′→3′)	Reverse primer (5′→3′)	Product length
14433	GAPDH	AGGTCGGTGTGAACGGATTTG	TGTAGACCATGTAGTTGAGGTCA	123bp
16193	il-6	AACGATGATGCACTTGCAGA	GAGCATTGGAAATTGGGGTA	283bp
21872	ZO-1	GAGCGGGCTACCTTACTGAAC	GTCATCTCTTTCCGAGGCATTAG	75bp
17831	Muc2	ATGCCCACCTCCTCAAAGAC	GTAGTTTCCGTTGGAACAGTGAA	101bp
15930	IDO-1	CAAAGCAATCCCCACTGTATCC	ACAAAGTCACGCATCCTCTTAAA	129bp
56720	TDO-2	AGGAACATGCTCAAGGTGATAGC	CTGTAGACTCTGGAAGCCTGAT	156bp
23923	KAT2	ATGAATTACTCACGGTTCCTCAC	AACATGCTCGGGTTTGGAGAT	137bp
17254	SLC3A2	ACGGTGTGGATGGTTTCCAAT	TCCCTGCAATCAAAAGCCTGT	120bp
20539	SLC7A5	CAGCTCCCTGAGTATGAAAGC	CCATTCCAGTAGACACCCCTTC	206bp
14964	H2-D1	TCCGAGATTGTAAAGCGTGAAGA	ACAGGGCAGTGCAGGGATAG	204bp
12258	SerpinG1	ATCCAAAGGTGTCACTTCTGTG	GCGGATCTTATGGTTGGTGTTC	184bp
14469	GBP2	CTGCACTATGTGACGGAGCTA	GAGTCCACACAAAGGTTGGAAA	115bp
14962	CFB	TGCTATGATGGTTACGTTCTCCG	TCCCAATAGGAATACCGGGATT	130bp
15040	H2-T23	AGAGTAACGACGAATCTCACACG	CTTGCAGGTATGCCCTCTGTT	225bp
20194	S100a10	TGGAAACCATGATGCTTACGTT	GAAGCCCACTTTGCCATCTC	182bp
13730	EMP1	TGAAGATGCTATCAAGGCAGTG	CTGGAACACGAAGACCACAAG	85bp
19288	PTX3	CGCAGGTTGTGAAACAGCAAT	GGGTTCCACTTTGTGCCATAAG	171bp
14131	CD16	AATGCACACTCTGGAAGCCAA	CACTCTGCCTGTCTGCAAAAG	79bp
14130	CD32	ATGGGAATCCTGCCGTTCCTA	CCGTGAGAACACATGGACAGT	66bp
12519	CD80	TGCTGCTGATTCGTCTTTCAC	GAGGAGAGTTGTAACGGCAAG	102bp
12524	CD86	CTGGACTCTACGACTTCACAATG	AGTTGGCGATCACTGACAGTT	131bp
17533	CD206	CTCTGTTCAGCTATTGGACGC	CGGAATTTCTGGGATTCAGCTTC	132bp
12655	YM-1	CAGGTCTGGCAATTCTTCTGAA	GTCTTGCTCATGTGTGTAAGTGA	197bp
21803	TGF-β	CTTCAATACGTCAGACATTCGGG	GTAACGCCAGGAATTGTTGCTA	142bp

### Enzyme-linked immunosorbent assay

For extracting total protein, the liver was homogenized in a mixture of RIPA buffer (P0013C, Beyotime, Shanghai, China), 2% phosphatase inhibitor (P1081, Beyotime, Shanghai, China) and 1% phenylmethanesulfonyl fluoride (PMSF, ST506, Beyotime, Shanghai, China). Following centrifugation at 14,000 r/min for 15 minutes at 4°C, supernatants were collected. The level of il-10 (EK0417, Boster, Wuhan, China) in colon was quantified by using commercial ELISA kit according to the manufacturer’s instructions. The detection limit of the kit is 15.6-1000 pg/ml for il-10.

### Disease activity index evaluation

As previously described, body weight loss, stool consistency and bleeding were recorded each day to determine the Disease Activity Index (DAI) (48). The scores were quantified as follows: weight loss: 0 (no loss), 1 (1-5%), 2 (5-10%), 3 (10-20%), and 4 (>20%); stool consistency: 0 (normal), 2 (loose stool), and 4 (diarrhea); and bleeding: 0 (no blood), 1 (Hemoccult positive), 2 (Hemoccult positive and visual pellet bleeding), and 4 (gross bleeding, blood around anus). DAI for each mouse was calculated by adding together the scores for weight loss, stool consistency and fecal blood.

### LC-MS/MS analysis

To detect the concentrations of Trp, Kyn and Kyna in serum and cerebral cortex, LC-MS/MS analysis was conducted according to Wang et al. with slight modifications (49). In brief, the analysis was performed using Orbitrap Q-Exactive Plus mass spectrometer (Thermo Fisher Scientific, Bremen, Germany) connected with Vanquish UHPLC System (Thermo Fisher Scientific, Bremen, Germany). Chromatographic separation was achieved on the ACQUITY UPLC BEH C18 column (100 mm × 2.1 mm, 1.7 μm, Waters Corporation, Milford, MA, USA). A linear gradient mobile phase containing 0.1% formic acid water (A) and acetonitrile (B) was programmed as follows: 0–0.5 min, 95% A; 0.5–5 min, 95–20% A; 5–6 min, 20% A; 6–6.1 min, 20–95% A and 6.1–8 min, 95% A (flow rate: 0.4 mL/min). For the electrospray ionization quantitative analysis, the positive ion mode with parallel reaction monitoring was utilized. The detailed MS ionization source conditions included spray voltage at 2800 V; Capillary temperature at 325°C; sheath gas flow rate at 35; aux gas flow rate at 15; sweep gas flow rate at 0; aux gas heater temperature at 350°C. Data were processed by using the Thermo Xcalibur software (Thermo Fisher Scientific, Waltham, MA, USA).

### Western blot analysis

Total protein concentration in liver was determined with a BCA Protein Quantification Kit (E112-02, vazyme, Nanjing, China). After boiling denaturation, total protein (35 μg) was separated by 10%~12% SDS-PAGE, then transferred to PVDF membranes (ISEQ00010, Millipore, Billerica, MA, USA). The membranes were blocked with 5% skim milk for 2 h at room temperature and then incubated (overnight at 4°C) with the following primary antibodies: Rabbit anti-indoleamine 2,3-dioxygenase 1 (IDO-1, 1:1000, 13268-1-AP, Proteintech, Wuhan, China), Rabbit anti-TDO-2 (1:500, 15880-1-AP, Proteintech, Wuhan, China), Rabbit anti-AADAT (KAT2, 1:500, A13090, ABclonal, Wuhan, China) and Rabbit anti-GAPDH (1:8000, ET1601-4, Huabio, Hangzhou, China). Then the HRP conjugated goat anti-rabbit IgG was incubated for 2 h at room temperature. The protein bands were visualized by enhanced chemiluminescence (ECL) reagent (P90720, Millipore, USA) and Gel Image System (Bio-Rad, Hercules, CA, USA). Target signals were analyzed by using Image J software (NIH, Bethesda, MA, USA).

### Bioinformatics analysis

IDO-1 gene expression levels of patients with IBD (GDS1615, GDS4365, GDS3119 and GDS3268) and of mouse colitis models (GDS3859) were downloaded from the NCBI GEO database.

### 16S rRNA sequencing

The genomic DNA was extracted from the fecal samples by using the PowerSoil DNA Isolation Kit (MOBIO, Carlsbad, CA, USA). The V3~V4 hypervariable regions of bacterial 16S rRNA gene were amplified with the primers 341F (5′-CCTACGGGNGGCWGCAG-3′) and 806R (5′-GGACTACHVGGGTATCTAAT-3′). The target fragments of PCR amplified products were recovered, and then the amplified products of PCR were quantified by fluorescence. The qualified sequencing library was used for Illumina MiSeq pyrosequencing analysis.

### Statistical analysis

Using the SPSS 22.0 software (IBM SPSS Statistics, Armonk, New York, USA), normality was evaluated by the Shapiro-Wilk test. Differences between the two groups were analyzed by independent samples T test and Mann-Whitney U test respectively for parametric values and nonparametric values. Differences among three groups were analyzed by one-way ANOVA with *post hoc* comparisons of Bonferroni and Kruskal-Wallis test for parametric values and nonparametric values. Values were shown as mean ± SEM. P < 0.05 was considered statistically significant between groups (*P < 0.05, **P < 0.01, ***P < 0.001).

## Data availability statement

The data presented in the study are deposited in the SRA database repository, accession number PRJNA916349.

## Ethics statement

The animal study was reviewed and approved by Animal Ethics Committee of Jiangnan University.

## Author contributions

Y-QS and L-PZ: conception and design of study. L-PZ, JW, WQ, YZ, HH, G-YN, TL and S-BH: acquisition of data. L-PZ, Y-QS, W-JZ, CC and C-MQ: analysis and interpretation of data. L-PZ and Y-QS: writing and review of the paper. All authors contributed to the article and approved the submitted version.
